# Supplementary figures and images for: Infant transmitted/founder HIV-1 viruses from peripartum transmission are neutralization resistant to paired maternal plasma
Source: PLoS Pathog. 2018 Apr 19;14(4):e1006944. doi: 10.1371/journal.ppat.1006944 (PMC5908066; doi:10.1371/journal.ppat.1006944)

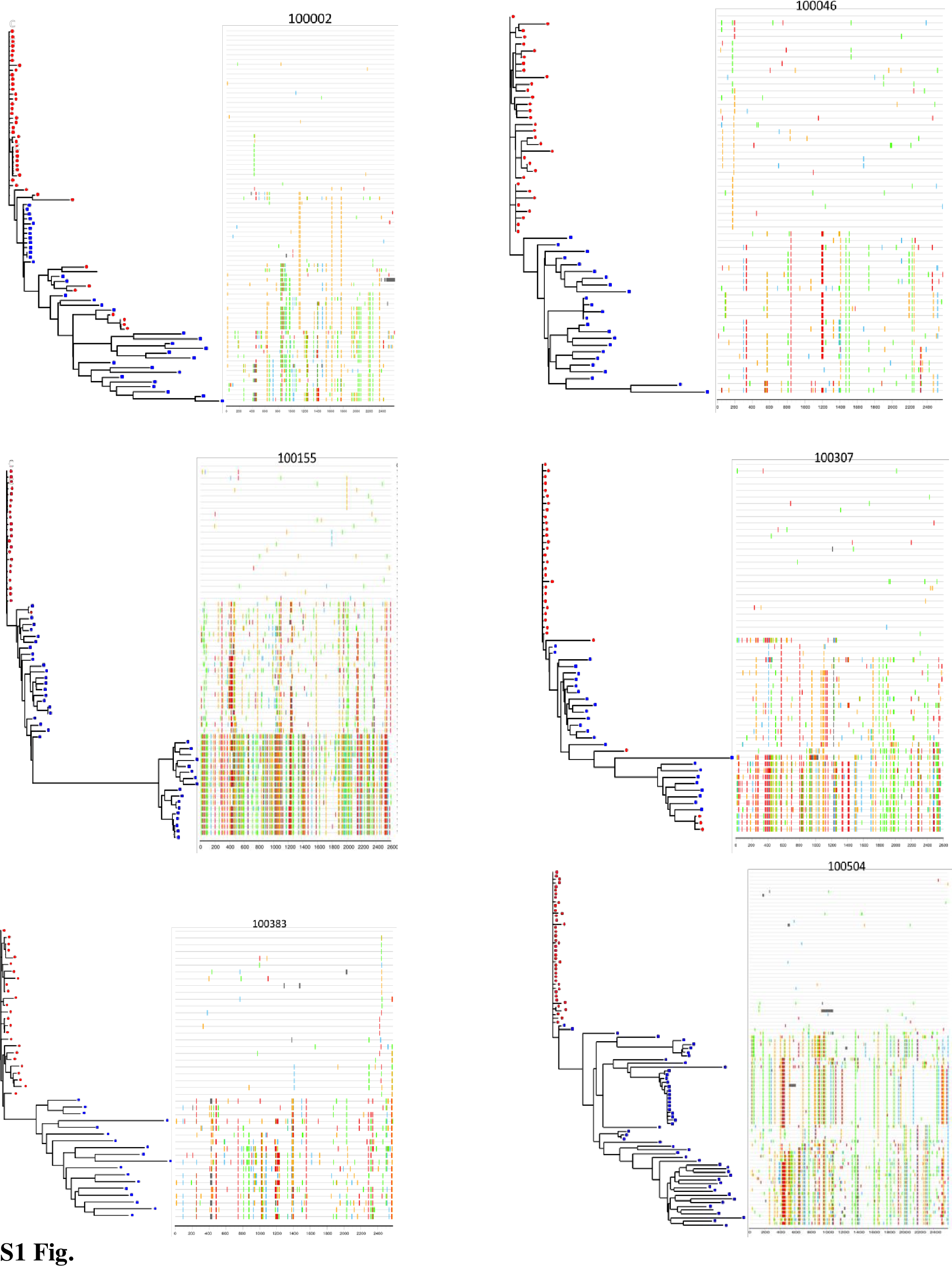

Supplement: S1 Fig — Infant sequences are labeled in red circles and maternal sequences are labeled in blue squares. (TIF) [file ppat.1006944.s001.tif]

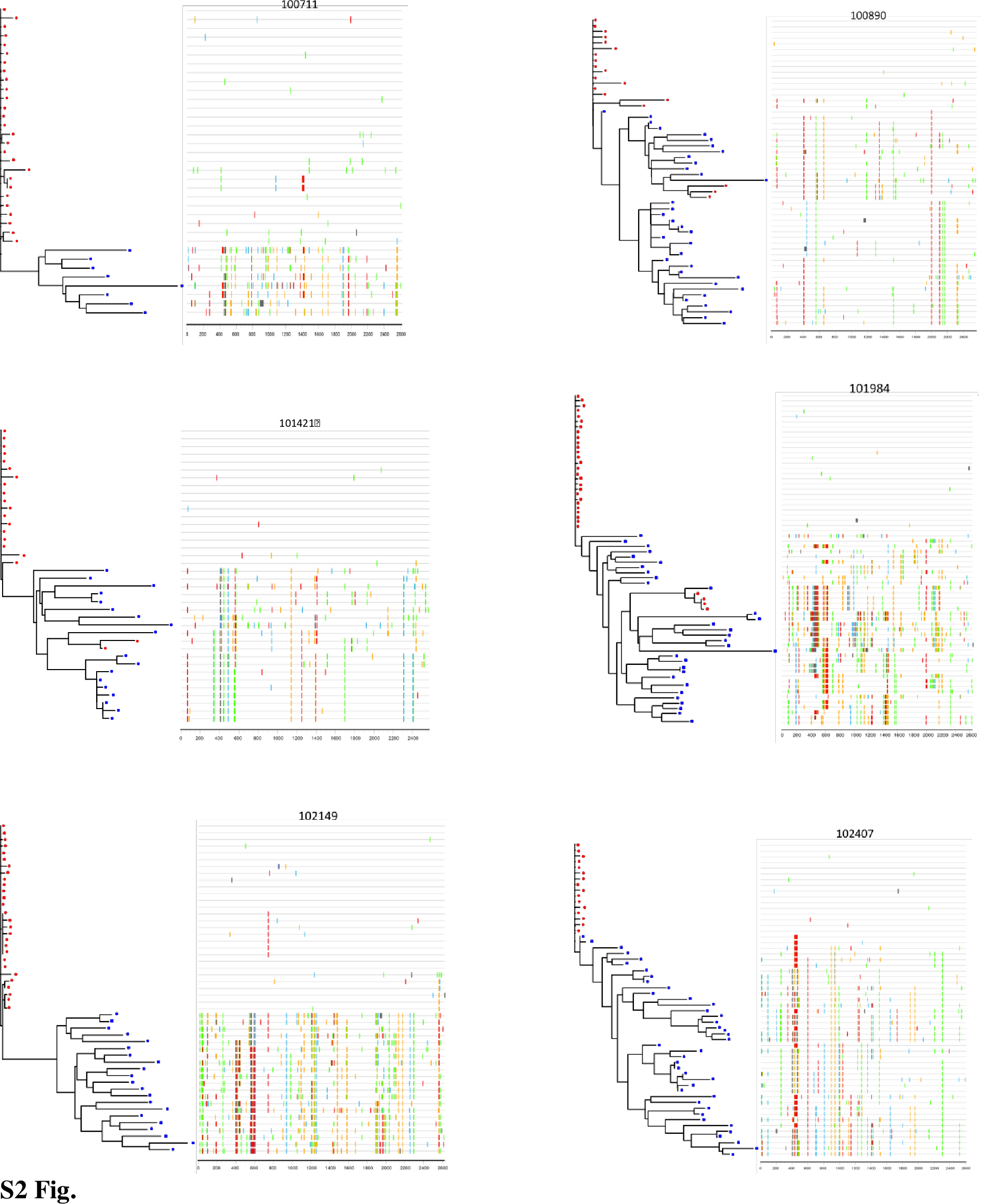

Supplement: S2 Fig — Infant sequences are labeled in red circles and maternal sequences are labeled in blue squares. (TIF) [file ppat.1006944.s002.tif]

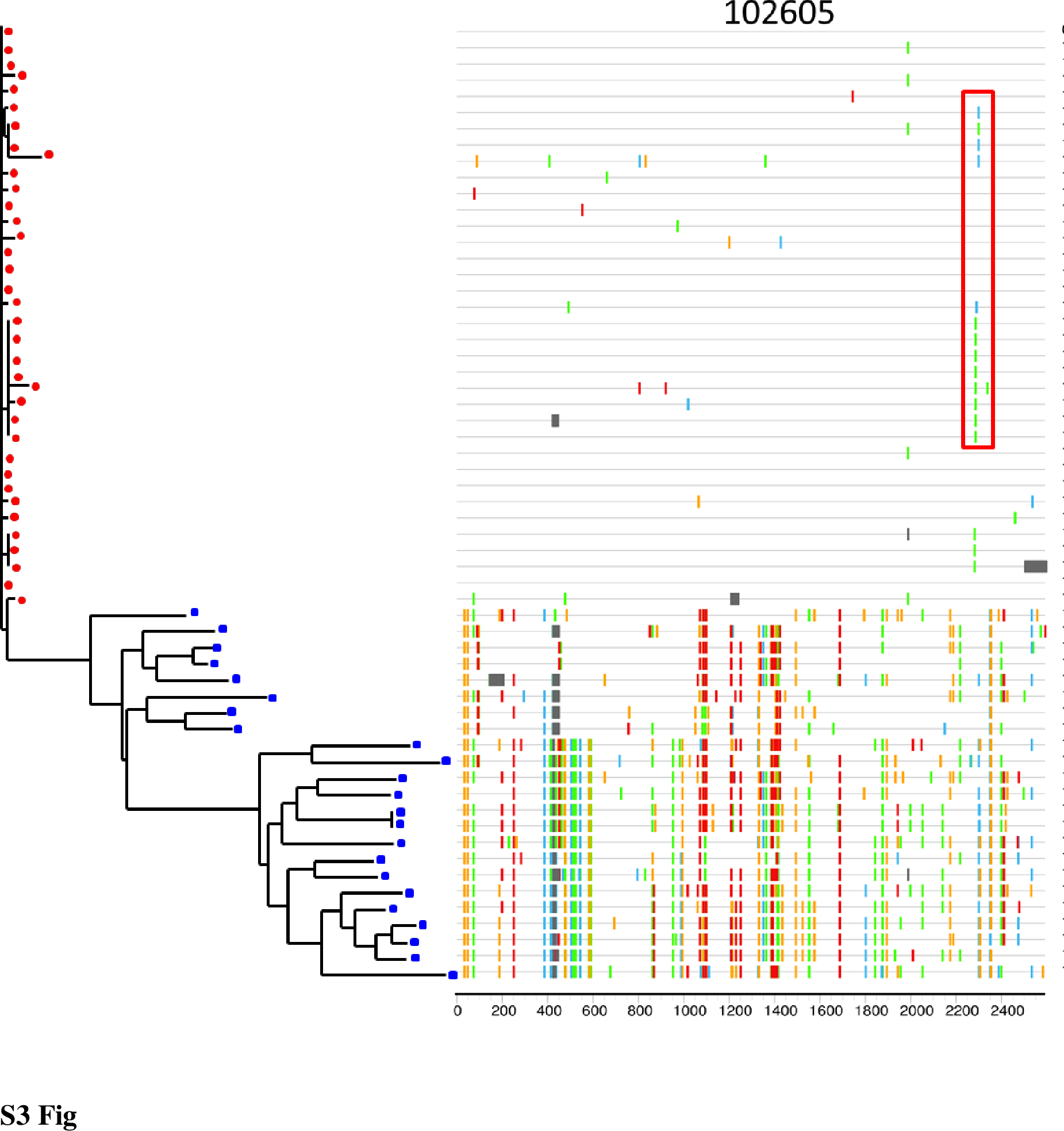

Supplement: S3 Fig — Infant sequences are labeled in red and maternal sequences are labeled in blue. The non-random accumulation of synonymous mutations in the infant (which caused the Poisson Fitter analysis to fail) is evident on the right as marked by red box. (TIF) [file ppat.1006944.s003.tif]

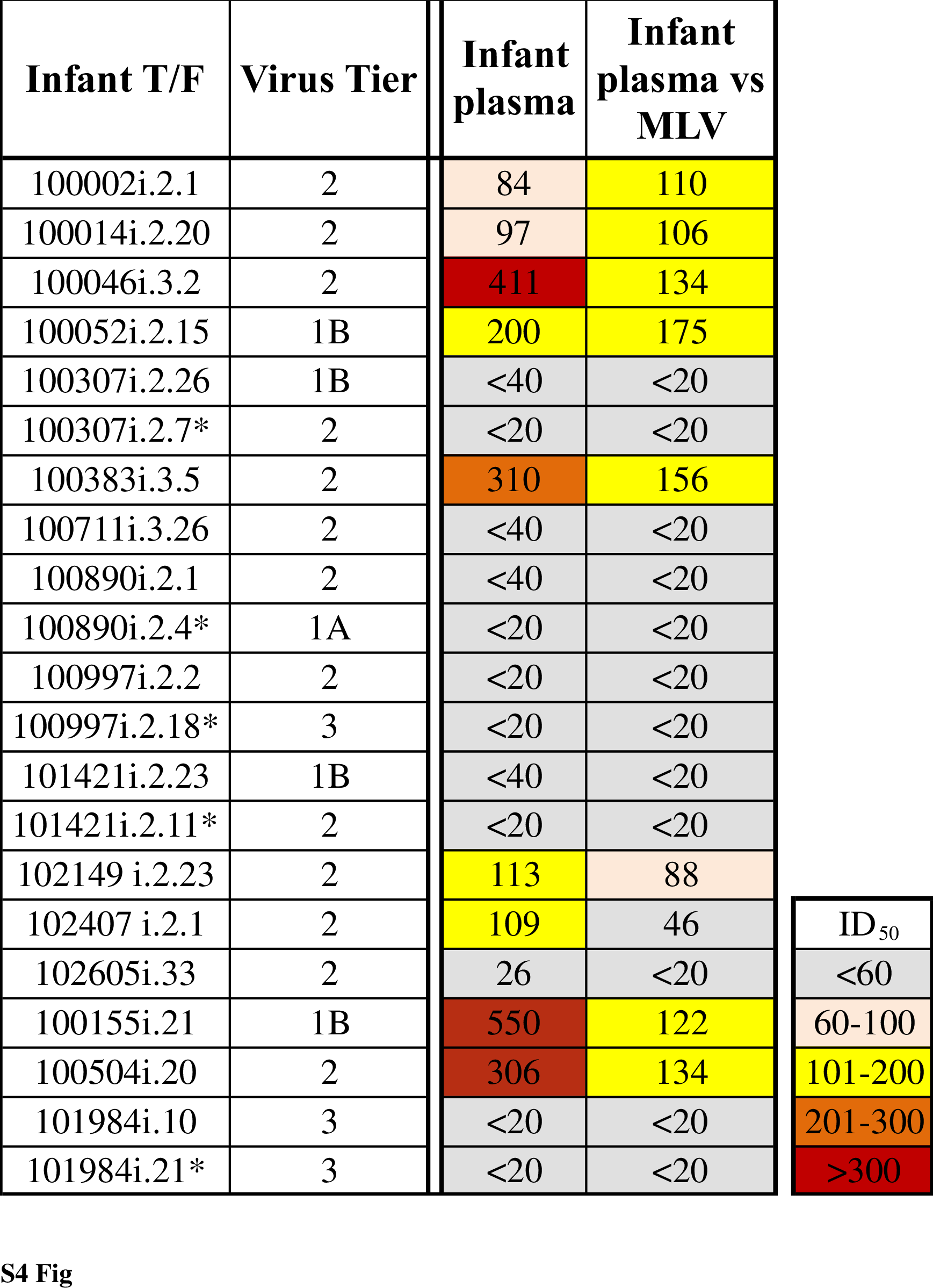

Supplement: S4 Fig — Dark colors represent viruses that were easily neutralized. The second T/F viruses are indicated with asterisks. (TIF) [file ppat.1006944.s004.tif]

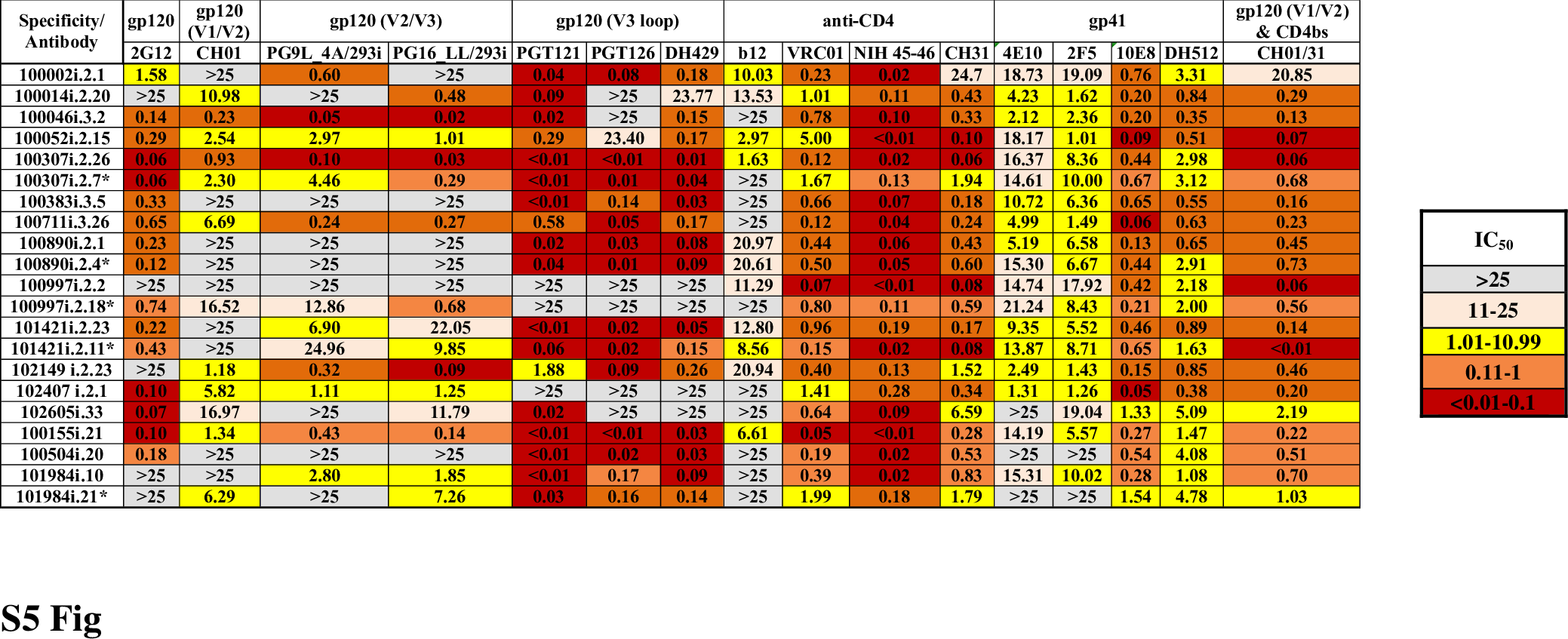

Supplement: S5 Fig — Dark colors represent viruses that were easily neutralized. The second T/F viruses are indicated with asterisks. (TIF) [file ppat.1006944.s005.tif]

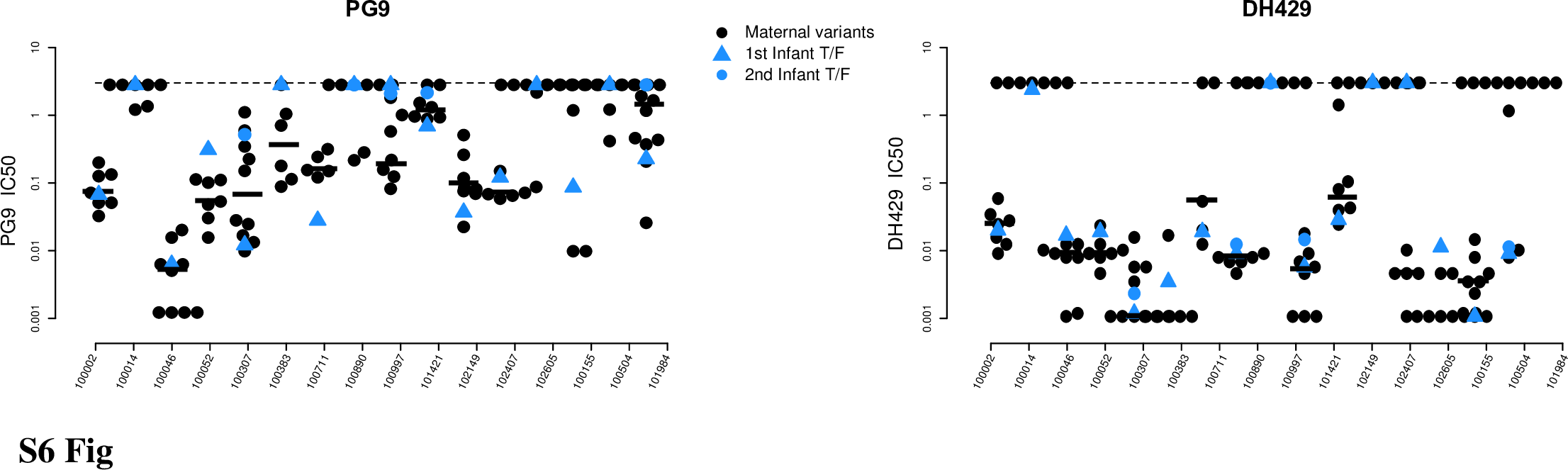

Supplement: S6 Fig — IC50 of maternal non-transmitted variants (black dots) and infant T/F viruses (blue triangles and circles) to two antibodies PG9 and DH429. Black horizontal lines represent the median of the IC50 of maternal and infant sequences. Dashed lines represent the detection limit. (TIF) [file ppat.1006944.s006.tif]

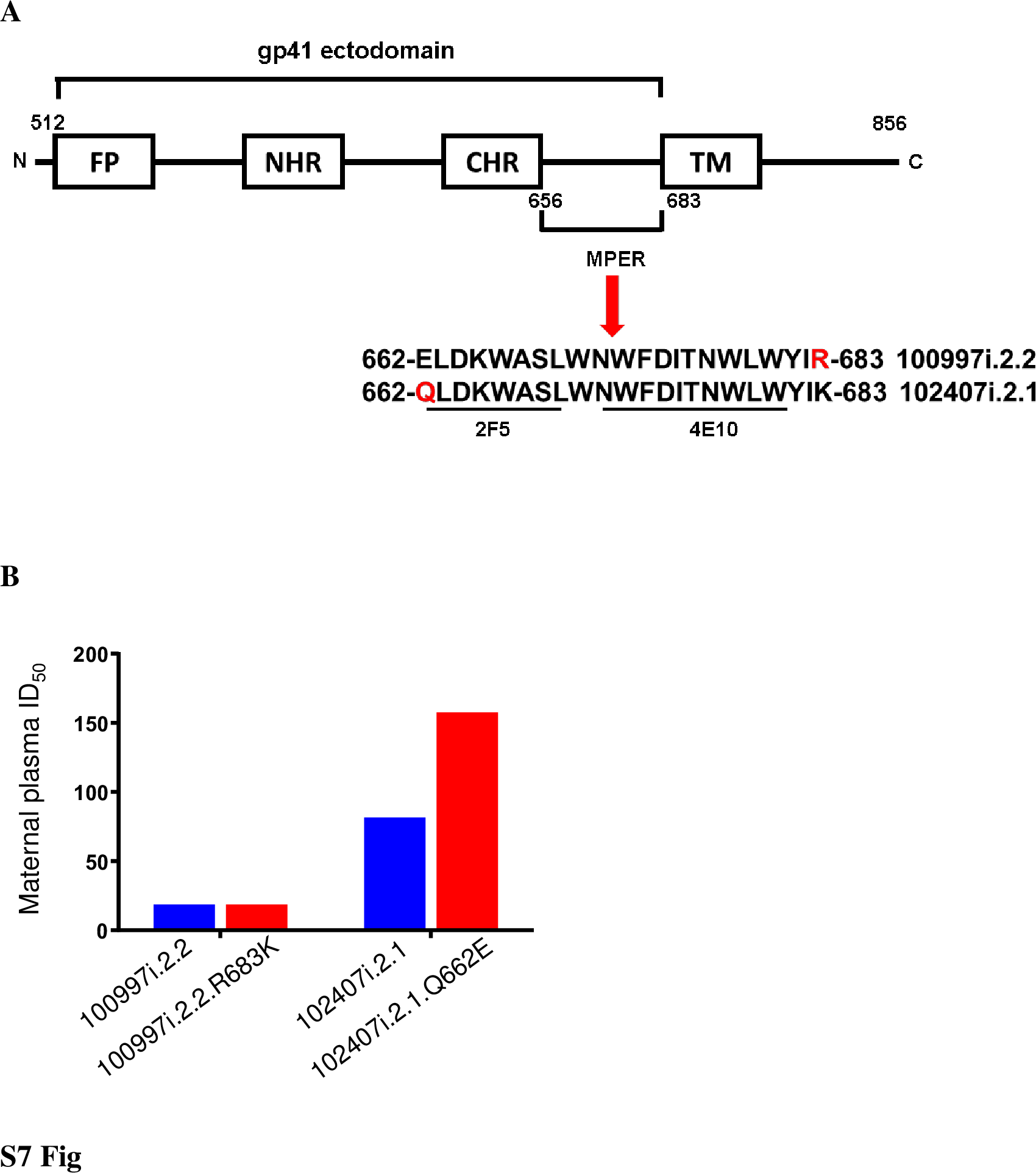

Supplement: S7 Fig — (A) Schematic of gp41 region indicates the position of mutated amino acids mutated. (B) Neutralization sensitivity of infant T/F viruses from 100997i and 102407i and their mutants to paired maternal plasma. ID50 of infant T/F variants (blue bars) and their point mutants (red bars). E662 and K683 are the signature sequence sites in the MPER region of HIV-1 envelope gene. FP = Fusion Peptide, NHR = N-terminal heptad repeat, CHR = C-terminal heptad repeat, MPER = Membrane Proximal External Region. (TIF) [file ppat.1006944.s007.tif]
